# Short-Term Effects of 3D-Printed Occlusal Splints and Conventional Splints on Sleep Bruxism Activity: EMG–ECG Night Recordings of a Sample of Young Adults

**DOI:** 10.3390/jcm13030776

**Published:** 2024-01-29

**Authors:** Andrea Bargellini, Elena Mannari, Giovanni Cugliari, Andrea Deregibus, Tommaso Castroflorio, Leila Es Sebar, Gianpaolo Serino, Andrea Roggia, Nicola Scotti

**Affiliations:** 1 Department of Surgical Sciences, Specialization School of Orthodontics, Dental School, University of Torino, 10126 Torino, Italy; bargelli@ipsnet.it (A.B.); andrea.deregibus@unito.it (A.D.); tommaso.castroflorio@unito.it (T.C.); 2 Department of Surgical Sciences, Gnathology Unit, Dental School, University of Torino, 10126 Torino, Italy; 3 Department of Surgical Sciences, Dental School, University of Torino, 10126 Torino, Italy; elena.mannari@edu.unito.it (E.M.); andrea.roggia@gmail.com (A.R.); 4 Department of Medical Sciences, University of Torino, 10126 Torino, Italy; giovanni.cugliari@unito.it; 5 Department of Applied Science and Technology, Politecnico di Torino, 10129 Turin, Italy; leila.essebar@polito.it; 6 Department of Mechanical and Aerospace Engineering, Politecnico di Torino, 10129 Turin, Italy; gianpaolo.serino@polito.it; 7 PolitoBioMedLab, Politecnico di Torino, 10129 Turin, Italy; 8 Department of Surgical Sciences, Restorative Dentistry Unit, Dental School, University of Torino, 10126 Torino, Italy

**Keywords:** sleep bruxism, 3D, occlusal splint, custom appliance

## Abstract

(1) **Background**: This study aims to compare the effects of 3D-printed splints and conventional manufactured splints on sleep bruxism (SB) EMG activity. (2) **Methods**: Twenty-six patients (19 M, 7 F, 25.8 ± 2.6 years) were randomly allocated to a study group (3D splints) and a control group (conventional manufactured splints) and followed for a period of three months with night EMG–ECG recordings. Samples of the involved materials were analyzed for nanoindentation. The outcomes of interest considered were the overall SB index, the total amount of surface masseter muscle activity (sMMA), and general and SB-related phasic and tonic contractions. A statistical evaluation was performed with a confidence interval (CI) between 2.5% and 97.5%. (3) **Results**: Differences between groups with OAs were observed for general tonic contraction (*p* = 0.0009), while differences between recording times were observed for general phasic contractions (*p* = 0.002) and general tonic contractions (*p* = 0.00001). Differences between recording times were observed for the total amount of sMMA (*p* = 0.01), for general phasic contractions (*p* = 0.0001), and for general tonic contractions (*p* = 0.000009) during night recordings without OAs. (4) **Conclusions**: Three-dimensional splints seem to have a higher impact on SB-related electromyographic activity but not on the overall sleep bruxism index. The more regular surfaces offered by 3D splints could be related to phasic contraction stabilization.

## 1. Introduction

According to the American Academy of Sleep Disorders (AASM), sleep bruxism (SB) is defined as a sleep-related movement disorder characterized by simple, often stereotyped movements occurring during sleep [1]. SB is not a movement disorder or a sleep disorder in otherwise healthy individuals, and it is characterized by involuntary phasic (rhythmic) or tonic (sustained) motor activity in the masticatory muscles (e.g., the masseter or the temporalis) during sleep [2,3]. Considerations of the physiological mechanisms beneath SB have shown that they are characterized by tachycardia, often followed by bradycardia, and can occur with or without EEG desynchronization [4]. These events are similar to the physiological sequences involved in rhythmic masticatory muscle activity in SB (RMMA/SB), which consist of the following activities: an increase in sympathetic activity before the RMMA/SB onset (−4 to −8 min) [5], followed by an increase in EEG activity (cortical arousal) (−4 s; tachycardia occurs 1 s before RMMA/SB [6,7], followed by an increase in the respiratory amplitude concomitant with RMMA/SB onset [8]. Further, SB is associated with significant increases in both systolic and diastolic blood pressure (BP) [9].

RMMA may lead to an imbalance of the stomatognathic system, involving the temporomandibular joints (TMJs) and their structures in the long term [10,11,12,13]. Rubin et al. and Wieckiewicz et al. reported that it is still questionable whether sleep bruxism is related to clinical signs of temporomandibular disorders (TMDs) [14,15]: in the literature, there have been authors reporting that there is a statistically significant correlation between TMDs and SB [16], as well as authors who have reported that this correlation is not statistically significant [17]. This may be due to different diagnostic criteria and study designs. Also, Topaloglu-Ak et al. determined that there is a significant association between negative sleeping habits and SB, TMDs, and dental caries [17].

Recent studies have also considered that the serotonin neurotransmission pathway may be involved in SB pathogenesis [18]. Serotonin (5-hydroxytryptamine, 5-HT) is a monoamine neurotransmitter of the central nervous system that is synthesized from tryptophan obtained from dietary sources [19]. During the synthesis, tryptophan is converted to 5-hydroxytryptophan (5-HTP) [20] through biopterin-dependent monooxygenation catalyzed via tryptophan hydroxylases 1 and 2 (TPH1 and TPH2), and then 5-HTP is decarboxylated via aromatic l-amino acid decarboxylase (DDC) to 5-HT [21]. However, the cause of the decreased serotonin levels in patients with severe SB is not known yet.

SB has been treated with different therapeutic approaches, such as oral appliance therapy (OAT) with stabilization splints, cognitive behavioral therapy (CBT), biofeedback therapy (BFT), and pharmacological therapy [22,23]. To date, the approach to SB has mainly focused on reducing SB’s detrimental effects on the stomatognathic system [24,25,26], and oral appliances (OAs) seem to be the standard reference [27]. Three-dimensional technology has been spreading in all dentistry fields since the early 1980s [28,29], and to date, a digital workflow is commonly applied from prosthodontics to orthodontics [30,31,32]. This technique has been applied even for OAs in the gnathological field with good results of accuracy and precision [33]. Over time, different materials have been involved in OA manufacturing in place of acrylic resin, such as a light-cured composite that, in preliminary studies, was preferred in terms of comfort by patients [34]. It is reasonable to question whether different approaches to OA manufacturing procedures, as well as different material selections, may thus influence the mechanical characteristics of such devices and whether they may consequently affect a neuromuscular response of the stomatognathic system. Moreover, the use of a simplified digital workflow tends to reduce the number of possible errors and distortions compared to traditional manufacturing, leading to a more precise casting in the laboratory and, therefore, to fewer occlusal adjustments on the dentist chair [35].

The aim of this randomized clinical trial was to answer the following questions:

- Do different OA fabrication techniques (i.e., 3D or traditional) influence SB?
- Do different OA materials influence perceived comfort during usage?
- Is there a difference in nanomechanical properties between the materials used to produce the two devices?

## 2. Materials and Methods

### 2.1. Study Design

The data reported in this investigation were gathered from a 2-arm parallel-group randomized controlled trial comparing SB index, sleep-time masticatory muscle activity (sMMA), and physiological and SB-related electromyographic contractions (i.e., tonic and phasic) between a group of subjects wearing a traditionally manufactured Michigan maxillary splint (control) and a group of patients wearing a 3D-printed Michigan maxillary splint (study). This trial was conducted following the Consolidated Standards of Reporting Trials (CONSORT) extension for pragmatic clinical trials [36]. Ethical approval was obtained from the Committee of the Research Department of the University of Torino, Italy (ethical approval #0089207). This trial was registered at ISRCTN.com (ISRCTN: ISRCTN91976427). All subjects provided their informed consent and signed an informed consent form before their enrollment in the study, and they were aware of the possibility of withdrawing from the study at any time.

### 2.2. Participants

To conduct this study, a total amount of 40 patients (26 M and 14 F; mean age: 26 ± 2.5 years) were randomly selected from a pool of patients at the Gnathology Unit of the University of Torino (Italy) with validated SB diagnosis conducted using a dedicated EMG–ECG holter (Bruxoff^®^, OT Bioelettronica, Torino, Italy) [37,38,39,40]. The patients who enrolled in this study had to fulfill the following inclusion criteria: (1) permanent dentition, (2) the absence of medications, (3) the absence of previous and/or active SB treatments, (4) the absence of periodontal disease evaluated according to the simplified oral hygiene index [41], (5) no medical history of neurological, mental, or sleep disorders, (6) an instrumental diagnosis of SB with at least 2 or more episodes per hour of sleep, and (7) no other underlying sleep disorders (such as obstructive sleep apnea or neurological sleep disorders) [42]. The exclusion criteria comprised the following: (1) active periodontal disease, (2) extended implant rehabilitations (>2 implants per arch), (3) active dental interventions (i.e., extractions, untreated caries, and prosthetic interventions with modifications of the occlusal plane), (4) missing teeth (>2 per arch), and (5) active SB treatments with OAs. After dropouts’ removal, data recorded from 26 patients (19 M and 7 F; mean age: 25.8 ± 2.6 years) were analyzed for the study as follows: 12 patients in the study group (7 M and 5 F; mean age: 26.7 ± 2.9 years) and 14 in the control group (12 M and 2 F; mean age: 25.4 ± 2.5 years). All data were collected and stored at the Orthodontics Unit of the Dental School of the University of Torino.

### 2.3. Randomization and Blinding

The randomization procedure consisted of a two-stage procedure in which subjects who entered the trial were first grouped into strata according to clinical features that could have influenced the outcomes: age and sex. No statistically significant differences in age and sex were shown after a Wilcoxon test between the groups. The patients’ assignment to the study or control group was randomly conducted using a computer-generated randomized table of numbers created by a statistician (G.C.). Individual and sequentially numbered index cards with random assignments were prepared, folded, and placed in sealed, opaque envelopes. The patients were blinded to the treatment. The clinicians’ blinding was not possible.

### 2.4. Outcome Measures

The outcomes of interest considered were the overall SB index, the total amount of surface masseter muscle activity (sMMA), and general and SB-related phasic and tonic contractions. A statistical evaluation was performed with a confidence interval (CI) between 2.5% and 97.5%.

### 2.5. Sample Size Calculation

The sample size calculation was based on data relating to the SB index (number of SB events per hour of sleep) estimated during screening in the full sample (40 subjects). Dropouts were included in the statistical analysis as an intention-to-treat (ITT) analysis. The mean ± SD of the SB index was 5.75 ± 3.96 SB episodes per hour of sleep. A 50% increase/decrease in the SB index was considered clinically relevant with an assumed SD of 3.96 and 80% power at the 5% significance level, which achieved the required sample size of 12 subjects per group.

### 2.6. Statistical Analysis

The normality assumption of the data was evaluated with the Shapiro–Wilk test [43]; the homoscedasticity and autocorrelation of the variables were assessed using the Breusch–Pagan and Durbin–Watson tests [44,45]. A two-way analysis of variance (ANOVA) was performed to estimate the differences in the δ (Tn–T0) means between groups stratified by splint use (yes/no) during Bruxoff^®^ recordings. All analyses were adjusted for sex, age, and the duration of the test. Descriptive values were shown with a consideration of the main indicators of distribution and variability. The level of significance was set to *p* < 0.05. Statistical analyses were conducted using the R statistical package (version 3.5.3, R Core Team, Foundation for Statistical Computing, Vienna, Austria) [46].

### 2.7. Sensitivity Analysis

Descriptive values were shown with a consideration of the main indicators of distribution and variability. The level of significance was set to *p* < 0.05.

### 2.8. Experimental Section

Digital impressions were recorded for both groups with an iTero Element 5D (Align Technology, Inc., San Jose, CA, USA), and stereo-lithographic (STL) models were crafted with a computer-aided design and computer-aided manufacturing (CAD-CAM) 3D printer, a SolFlex 650 (VOCO^®^ GmbH, Cuxhaven, Germany).

The OAs used in the study were realized as follows:

(1) Study group: Splints were manufactured thanks to the 3D printer, a SolFlex 650 (VOCO^®^ GmbH, Cuxhaven, Germany), with an additive technique. The light-cured V-Print Splint resin (VOCO^®^ GmbH, Cuxhaven, Germany) was used for this purpose, classified as class IIa medical disposal [47] (medium risk, non-active disposal), biocompatible, tasteless, transparent, and with high resistance to abrasion (reported flexural strength: 75 Mpa). A 0.20 mm offset value was used for the additive technique, as suggested by Lo Giudice et al. [48]. After printing, all debris was removed with an ultrasonic bath of isopropyl alcohol, and the plates were dried with compressed air. Fifteen minutes after the final contact with isopropyl alcohol, the plates were post-cured on both sides with an OtoFlash-Polymerization Unit (VOCO^®^ GmbH, Cuxhaven, Germany): 3.5 min per side with a frequency of 10 flashes per second for a total amount of 2000 flashes. At the end of the process, all the plates were finished and polished by an expert dental technician of the Dental School, University of Torino, Torino (Italy).
(2) Control group: Splints were manufactured with a 1.25 mm thermoformed retentive base of polyethylenterephthalat–glycol copolyester (PET-G) (DURAN^®^ Scheu-Dental, Am Burgberg, Germany) (reported flexural strength: 69 Mpa) rebased with self-curing resin (Forestacry^®^, Forestadent Bernhard Förster GmbH Westliche, Pforzheim, Germany), classified as class I medical disposal [47] (non-critical and non-active disposal), biocompatible, tasteless, transparent, and with high resistance to abrasion (reported flexural strength: ≥50 Mpa). The plates were fabricated, finished, and polished by an external dental laboratory (GLOI Laboratorio Odontotecnico, Biella, Italy).

Both OAs were designed as Michigan maxillary splints with an increase in the occlusal vertical dimension (OVD) of at least 2 mm in the molar area (Maestro 3D software (www.maestro3d.com, accessed on 27 November 2023), AGE Solutions S.r.l, Pontedera, PI, Italy). Occlusion was checked with an 8 μm Bausch articulating paper (Bausch^®^, Nashua, NH 03062 USA) in order to let arches occlude evenly and uniformly.

All patients were instructed by expert clinicians (T.C. and E.M.) on the use and maintenance of their splints, with the recommendation to wear them every night for three months.

SB monitoring was performed for all patients with the Bruxoff^®^ device [37,38,39,40] (OT Bioelettronica, Torino, Italy) as follows: at OA delivery (T0), after 1 month since delivery (T1), and after three months since delivery (T2). Only during the T0, T1, and T2 stages were the patients asked to perform two different night recordings: one night wearing the splint and the following night without wearing it.

All the patients were instructed by expert clinicians (T.C., A.B., and E.M.) on the use of the Bruxoff^®^ device. The data were analyzed with dedicated software (Bruxmeter (version 2.0.2.4)^®^ OT Bioelettronica, Torino, Italy) by an expert clinician blinded to the study (A.B.).

At the T1 stage, the patients were asked to fill in a modified version of the Oral Health Impact Profile (OHIP-14) [49] to investigate their perceived comfort with different OAs (Table 1). The data were inserted into an Excel^®^ table by a researcher not blinded to the study (E.M.) for statistical analysis (Microsoft Corporation, Redmond, WA, USA).

### 2.9. Nanoindentation Protocol

Square-designed specimens (1 cm^2^ and 1 mm thick) were prepared by following the same procedure used to fabricate the OAs under investigation. The surfaces of the two specimens were properly polished and rinsed with isopropyl alcohol. The data were recorded by expert clinicians (N.S. and A.R.) and analyzed by an expert engineer in the field (G.S).

Nanoindentation tests were carried out, imposing a maximum indentation depth of 2000 nm. Forty-two indentations per specimen were performed in order to investigate the mechanical properties of an area of 16 mm^2^. The nanoindentation protocol was characterized by three phases. During the first step, the nanoindenter tip was brought in contact with the specimen surface. After the contact detection, the penetration rate of the indenter was set to 15 mN/s, controlling the load imposed by the indenter on the surface sample. When the set penetration depth was reached (2000 nm), the load was held constant for a period of 5 s. Finally, the indenter tip was retracted at the same rate as the loading phase.

The nanoindentation curves were analyzed using the Oliver and Pharr method [50] and following the ISO 14577-1:2015 [51] in order to obtain the nanoindentation modulus (E_IT_).

A one-way analysis of variance was performed with a level of confidence equal to 0.01 to verify the difference in the nanoindentation moduli (E_IT_) obtained for the two materials.

## 3. Results

**Figure 1:**
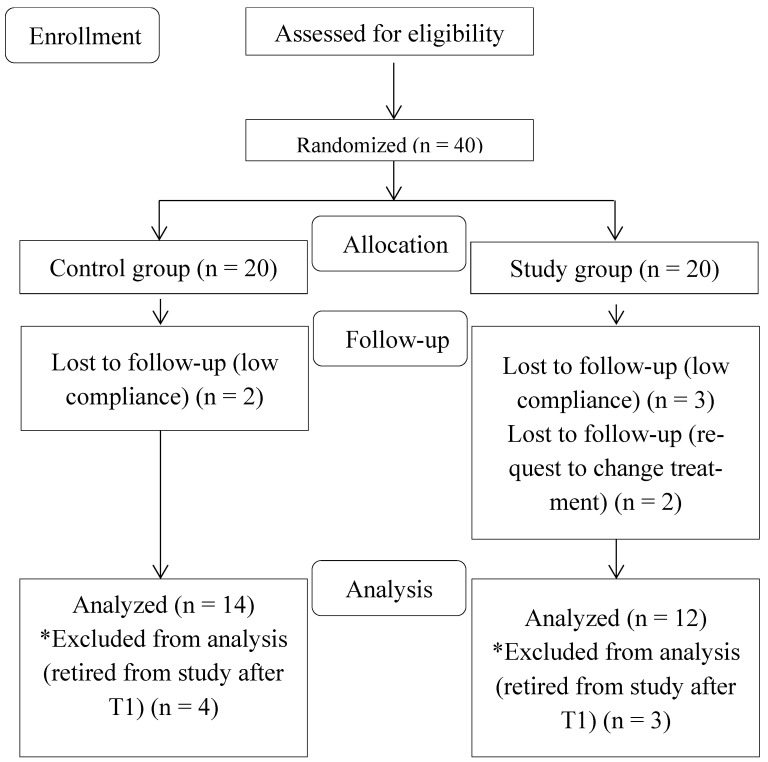
Consolidated Standards of Reporting Trials (CONSORT) diagram showing the flow of subjects in the study. Please note that * stands for retired patients from the study after T1.

A CONSORT diagram with subjects’ flow through the trial is shown in Figure 1. A total amount of 6287 min of sleep data were recorded. The mean sleep duration was 8.01 ± 1.2 h in the study group and 8.03 ± 1.4 h in the control group, with no differences between the groups. The mean SB index of the overall population was 5.4 ± 1.2 per hour of sleep. The patients in the study group showed 4.76 ± 1.8 SB episodes per night, and the control group showed 6.05 ± 2 SB episodes per night during three months of observation. The average values of outcome variables during the recording time (T0–T2) are reported in Table 2. The ANOVA results are reported in Table 3 and Table 4. At the end of the observation time of three months, the SB index was affected by neither traditional nor 3D-fabricated occlusal splints. Differences between the groups with OAs were observed for general tonic contraction (*p* = 0.0009), while differences between the recording times were observed for general phasic contractions (*p* = 0.002) and general tonic contractions (*p* = 0.00001). Differences between the recording times were observed for the total amount of sMMA (*p* = 0.01), for general phasic contractions (*p* = 0.0001), and for general tonic contractions (*p* = 0.000009) during night recordings without OAs.

### Nanoindentation

**Figure 2:**
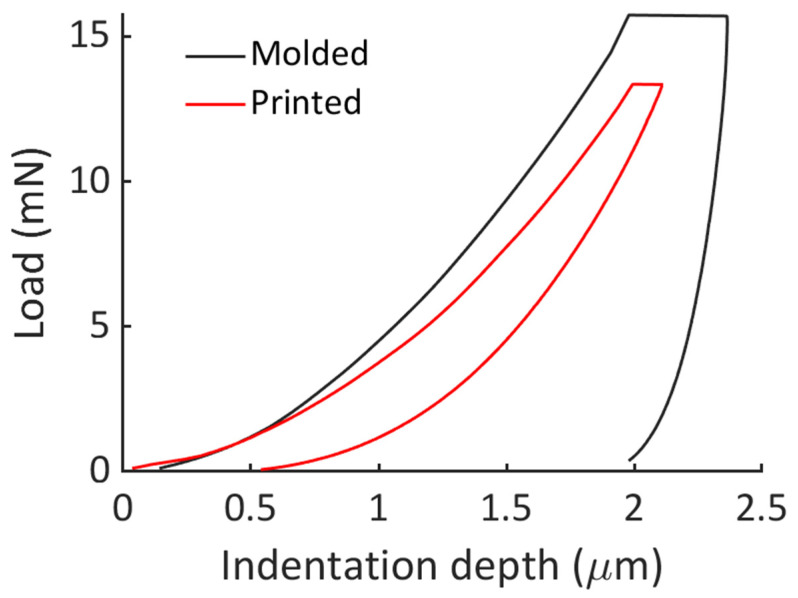
Representative nanoindentation curves obtained for molded and printed specimens.

Representative nanoindentation curves are reported in Figure 2. To reach the same indentation depth, the applied load on the molded specimen was higher compared to the printed specimen. Furthermore, the statistical analysis performed on the dataset of the nanoindentation modulus showed a significant difference between the elastic properties of the two materials (*p* = 0.0002).

**Figure 3:**
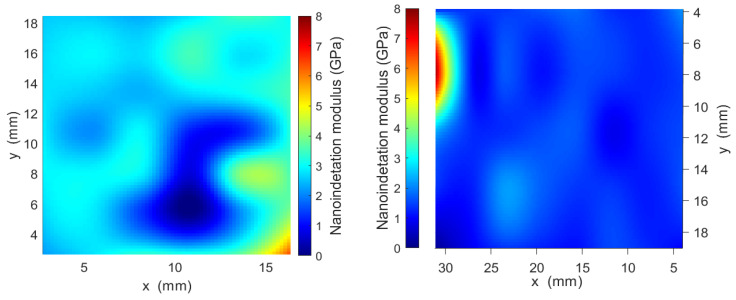
Color maps of nanoindentation moduli for molded (**on the left**) and printed (**on the right**) specimens.

**Figure 4:**
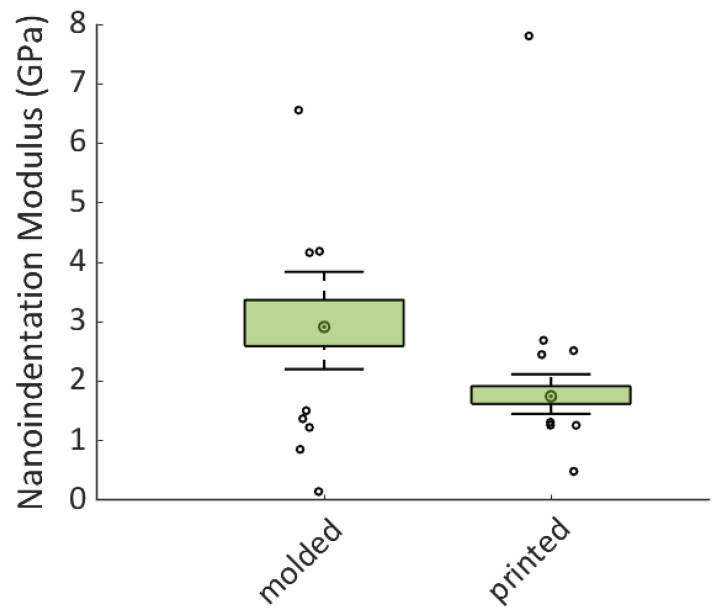
Dispersion of the nanoindentation modulus values reported through a box plot.

Box plot and color maps of the nanoindentation modulus are shown in Figure 3 and Figure 4, respectively. The spatial distribution and the dispersion of the elastic properties of the two materials were very different, indicating a significant difference between the two fabrication processes. The 3D-printed specimen was found to be non-homogeneous compared to the molded specimen. Indeed, the two color maps shown in Figure 4 corroborate the dispersion reported in the box plot of Figure 3. On the other hand, the molded specimen was found to be significantly stiffer than the printed one. As a matter of fact, while the printed specimen was found to be very homogeneous in terms of elastic properties, with a little spot where the elastic properties increased, the molded sample showed the opposite feature, with the spatial distribution of the elastic properties characterized by a predominance of higher values of the nanoindentation modulus compared to the lower ones measured in a smaller spot of the analyzed area.

## 4. Discussion

The present study aimed to investigate possible effects on the SB index of 3D-printed splints. Although some effects were observed on general EMG signals for three months, no influences on SB activity were found. Three-dimensional splints seem to better control general phasic contractions over time [52]; indeed, from the first to the third month of observation, the patients with 3D splints tended to maintain a stable trend, while the patients wearing traditionally manufactured splints showed a constant increase from T0 to T2. Thus, 3D splints could lead to an easier and faster adjustment in patients showing a high ratio of phasic contractions; on the other hand, traditionally manufactured splints should be avoided in these cases since their use increases these specific EMG signals in the first three months after delivery. Given general tonic contractions [5], statistically significant differences between the groups and the observation times were detected: despite a general increase for both groups after one month from delivery, tonic contractions tended to be lower for the patients wearing traditionally manufactured splints, and this trend reduced after three months, while it drastically increased for the patients wearing 3D splints. Traditionally manufactured splints should, then, be indicated for patients with higher levels of tonic contractions, while 3D splints should be avoided since their use tends to increase these EMG signals during the first three months after delivery. These effects should be related to the different flexural strengths of the involved materials: 3D splints fabricated with multiple layers of the same material (V-Print Splint resin (VOCO^®^ GmbH)) present a higher flexural strength (75 Mpa) than traditional splints fabricated with a thermoformed layer of polyethylenterephthalat–glycol copolyester (PET-G) (DURAN^®^ Scheu-Dental, Am Burgberg 20, 58642 Iserlohn, Germany) (reported flexural strength: 69 Mpa) rebased with self-cured resin (Forestacry^®^, Forestadent Bernhard Förster GmbH Westliche, Pforzheim, Germany) with lower flexural strength (>50 Mpa). It could be inferred that layers of a single hard resin should positively influence general phasic (i.e., brief and rhythmic) contractions, leading to a stabilization trend within one month after use, but they negatively affect tonic (i.e., sustained) contractions, leading to a detrimental and significant increase in their level, while thermoformed layers of PET-G covered with more elastic resins tend to positively affect general tonic contractions, but by contrast, they negatively influence general phasic contractions, leading to their constant increase for three months. The sMMA activity shows significant variations over time when OAs are not in use during night recordings. This effect could be explained by different behaviors of the EMG signals composing the overall sMMA index: tonic contractions maintained an increasing trend for the study group, while they decreased in the control group; phasic contractions remained stable in the study group, while they decreased in the control group. Generally, phasic contractions seem to be more influenced by the use and the interruption of splints with low flexural strength, while both tonic and phasic contractions tend to maintain an imprint from splints with high flexural strength. When in use, traditional splints reduce tonic contractions and increase phasic contractions; when not in use, both tonic and phasic contractions are reduced. When in use, 3D splints stabilize general phasic contractions and increase tonic contractions; when their use is interrupted, the effect on motor units seems to be imprinted since the effect on general phasic and tonic contractions remains the same: general phasic contractions remain stable and tonic contractions increase. Hence, patients with higher levels of general phasic contractions will take more time to adapt to their device if it is fabricated traditionally with resins with low flexural strength (>50 Mpa). A similar effect for traditionally manufactured splints was reported by Matsumoto et al. [53] in patients wearing occlusal splints at intermittent times; they reported significant reductions in nocturnal EMG events and duration immediately after splint delivery and after one month later when compared to a pool of patients wearing occlusal splints for 29 nights continuously. Patients wearing occlusal splints for an entire month experienced an immediate reduction after splint delivery and after one week, without any reduction at 2, 3, and 4 weeks. Unfortunately, no other indications of EMG signals were reported (i.e., phasic or tonic EMG signals); thus, no other conclusions can be added. These findings could be helpful for future studies related to the cost benefit for patients: since 3D splints entail higher manufacturing costs, the use of traditional splints for patients showing higher tonic contractions could be helpful both for clinical use and for making their application more affordable for patients.

To answer the second question related to the possible influences of different materials on perceived comfort, only self-reported improvement in patients’ quality of life and a perceived reduction in the night parafunction (questions 10 and 11 reported in Table 1) showed significant results: the patients wearing 3D-printed splints reported an improvement in their lifestyle in 64% of cases and referred to a perceived reduction of their parafunction in 63% of cases. These data suggest higher adaptation to 3D splints than to traditional ones if we consider the short-term effects on phasic and tonic contractions reported above. Anyway, these results reflect a perceived sensation and not the real trend of the instrumental data. As reported by Lobbezoo et al. [54], questionnaires are only able to indicate possible SB.

The mechanical properties of 3D-printed occlusal splints have been an area of active research in recent years, as the performance and durability of these splints depend on their material properties [55,56,57]. The present study results suggested that 3D-printed OAs had a lower nanoindentation modulus than the molded ones, suggesting lower mechanical properties. In the recent literature, several studies have investigated the mechanical properties of 3D-printed occlusal splints. A study by Cheah et al. [58] evaluated the mechanical properties of occlusal splints printed with two different 3D printing technologies, stereolithography (SLA) and digital light processing (DLP). The study found that the SLA-printed splints had higher flexural strength and wear resistance compared to the DLP-printed splints. Another study by Hwang et al. [59] investigated the mechanical properties of 3D-printed occlusal splints made from a biocompatible resin. The study found that the splints had a high degree of accuracy and precision, and they demonstrated good mechanical properties, including flexural strength and surface hardness. While most studies have suggested that 3D-printed occlusal splints have better mechanical properties than molded ones, there are some studies that have reported different findings, in accordance with the present study results. Li et al. [60] compared the mechanical properties of 3D-printed and traditionally polymerized occlusal splint materials and found that the traditionally polymerized splints had higher flexural strength values. Another study by Nayyer et al. [61] compared the wear resistance of 3D-printed and milled splints and found that the milled splints had better wear resistance. Paradowska–Starz et al. [62] pointed out that the long-term use of these resins must be taken into consideration, as well as aging’s influence on their structure: in their study, these authors stated that artificial aging has a deeply negative impact on 3D-printed splints, involving both the compressive modulus and tension of the material, and polishing was suggested to increase resin’s resistance to aging.

Overall, the literature suggests that 3D-printed occlusal splints can have good mechanical properties, but the performance can vary, depending on the printing technology and material used. It is important to note that the materials and printing/molding techniques used in each study may have differed, and these factors can have a significant impact on the mechanical properties of the resulting splints. Therefore, to answer the third question of this study, it is difficult to generalize about the strength of 3D-printed versus molded occlusal splints without considering the specific materials and techniques used in each case.

The uniformity of elasticity and hardness on the surface that meets the antagonistic teeth, on the other hand, should be considered when considering an OA. As shown in the color maps of Figure 3, the 3D-printed OAs seem to have a more uniform nanoindentation modulus than the molded ones. The better phasic contraction adjustment observed in patients wearing 3D-printed OAs during the study period may, therefore, be explained by the mechanical property pattern, which appears to be more regular over the device surface and results in a better and more consistent response to occlusal contact and sliding.

Outside the findings of this study, it is undoubted that the approach to SB should be carefully investigated with validated disposals to avoid overtreatments. The SB index, anyway, should be considered in association with all EMG activities to complete diagnoses and provide correct treatments. As stated by Lobbezoo et al., bruxism-related masticatory muscle activity should be assessed in its continuum, thus not only focusing on the raw number of bruxism events to correlate with clinical consequences [62]. It is not the number of bruxism events per se that represents a risk factor but, rather, the general level of EMG activity, which was found to be higher in temporomandibular disorder cases than in controls [63]. Such an approach is in line with the work of Greene et al. since the use of more technological and sophisticated investigation systems tends to be used to force treatments in non-symptomatic patients [64,65]; therefore, the use of occlusal splints in patients affected by SB and/or with a high number of EMG activities should be considered for symptomatic TMD patients.

### Limitations

There were two main limitations to the current study. First, the high number of dropouts led to a low number of participants. Second, the short period of observation reduced the power of this study. Another minor limitation could be related to the comparison between only two materials.

## 5. Conclusions

The overall SB index seems not to be affected in the first three months using different OAs fabricated with traditional or CAD-CAM techniques, while their short-term effects can be observed on general EMG signals. Perceived improvements in patients’ quality of life should be carefully considered since they could not evenly report the real physiological status observed with an instrumental investigation. The more regular surfaces achieved with 3D splints could be related to phasic contraction stabilization. Further studies conducted over a longer period with a higher number of participants are requested to improve the results obtained in this study.

## Figures and Tables

**Table 1:** The modified version of the Oral Health Impact Profile (OHIP-14) questionnaire.

Did you feel discomfort with your teeth when your splint was delivered?
2. If yes, how much did it hurt on a scale from 1 to 10?
3. I yes, which teeth hurt the most?
4. Could you wear your splint all night long?
5. If not, why?
6. Did you find an improvement in splint fit within one month of delivery?
7. How many nights did you wear your splint within one month of delivery?
8. If less than three nights a week, could you tell us why?
9. How much did your teeth hurt on a scale from 1 to 10 one month after splint delivery?
10. Did you find an improvement in your lifestyle one month after splint delivery (i.e., head and facial muscle tenderness, headache, or dental sensitivity)?
11. Did you perceive a reduction in your parafunction one month after splint delivery?

**Table 2:** Average values (standard deviation, SD) of outcome variables over each recording night per group with and without splints.

Outcome Variable	Group	Recording Nights (T0–T2)	Average Values (SD) with Splint	Average Values (SD) without Splint
SB index	3D splint	T0	4.8 (1.6)	5.1 (1.3)
		T1	6.1 (3)	7.3 (3.8)
		T2	3 (2.1)	5.4 (5.4)
	Control splint	T0	6 (2)	5.7 (2)
		T1	4.3 (2.2)	6.2 (2.4)
		T2	5.3 (3.5)	5.5 (3.5)
Total sMMA contractions	3D splint	T0	76.3 (50)	69.7 (43.7)
		T1	221.4 (134.4)	217 (147.6)
		T2	255.6 (117.2)	265.3 (156.6)
	Control splint	T0	84 (41.5)	87 (45.6)
		T1	150 (92)	212.8 (213.6)
		T2	129.1 (83)	155 (71)
Phasic sMMA contractions	3D splint	T0	20.8 (18.3)	26.7 (13.4)
		T1	60.4 (42.9)	61.3 (45.2)
		T2	60.2 (20.6)	60.4 (36)
	Control splint	T0	26.9 (14)	20.3 (11.5)
		T1	46.5 (37.5)	54.1 (62)
		T2	85.8 (144.4)	44.7 (26)
Phasic sMMA contractions, SB-related	3D splint	T0	6.9 (4.2)	6.1 (3.7)
		T1	10.2 (8.2)	11.8 (9.1)
		T2	4.3 (4.8)	6.7 (4.9)
	Control splint	T0	12.8 (6.1)	11.3 (4.7)
		T1	8.2 (7.6)	8 (5.9)
		T2	16 (32.9)	7 (5.6)
Tonic sMMA contractions	3D splint	T0	22.4 (20.8)	23.5 (19.6)
		T1	69.2 (51.6)	67.3 (37.3)
		T2	91.6 (51.3)	79 (50.6)
	Control splint	T0	20.2 (15.1)	22.6 (16.1)
		T1	42.7 (29.4)	64.7 (70.8)
		T2	31 (31.8)	38.2 (19.9)
Tonic sMMA contractions, SB-related	3D splint	T0	8.1 (5.1)	8.5 (4.3)
		T1	12 (7)	17.5 (11.5)
		T2	7.7 (5.5)	9.6 (8.4)
	Control splint	T0	10.4 (8.2)	9.8 (10.4)
		T1	9.6 (9)	8.7 (7.8)
		T2	19.3 (48.5)	8.5 (4.6)

SB: sleep bruxism. sMMA: surface masseter muscle activity. T0: baseline. T1: 1 month. T2: 3 months.

**Table 3:** Results of the two-way ANOVA test for night recordings with OAs.

Outcome Variable	Estimate	F Value	Pr (>F)
SB index	Group	0.918	0.340
	Time	1.659	0.196
Total sMMA contractions	Group	3.139	0.0797
	Time	0.613	0.5438
Phasic sMMA contractions	Group	0.276	0.600464
	Time	6.335	0.002646 **
Phasic sMMA contractions, SB-related	Group	2.965	0.0885
	Time	0.093	0.9114
Tonic sMMA contractions	Group	11.659	0.000952 ***
	Time	12.928	0.00001 ***
Tonic sMMA contractions, SB-related	Group	0.710	0.4015
	Time	0.465	0.6296

SB: sleep bruxism. sMMA: surface masseter muscle activity. *, significant at *p* < 0.05. **, *** number of asteriscs is indicative for statistical significancy

**Table 4:** Results of the two-way ANOVA test for night recordings without OAs.

Outcome Variable	Estimate	F Value	Pr (>F)
SB index	Group	0.113	0.737
	Time	1.885	0.158
Total sMMA contractions	Group	0.738	0.39247
	Time	4.501	0.01365 *
Phasic sMMA contractions	Group	0.145	0.70456
	Time	9.551	0.00017 ***
Phasic sMMA contractions, SB-related	Group	0.863	0.35535
	Time	2.158	0.12136
Tonic sMMA contractions	Group	1.802	0.18281
	Time	13.176	0.000009 ***
Tonic sMMA contractions, SB-related	Group	1.929	0.16823
	Time	2.350	0.10104

SB: sleep bruxism. MMA: surface masseter muscle activity. *, significant at *p* <0.05. *** number of asteriscs is indicative for statistical significancy

## Data Availability

Data is unavailable due to privacy restrictions.

## References

[1] Sateia M.J. (2014). International Classification of Sleep Disorders.

[2] Lobbezoo F., Ahlberg J., Raphael K.G., Wetselaar P., Glaros A.G., Kato T., Santiago V., Winocur E., De Laat A., De Leeuw R. (2018). International consensus on the assessment of bruxism: Report of a work in progress. J. Oral. Rehabil..

[3] Lavigne G.J., Khoury S., Abe S., Yamaguchi T., Raphael K. (2008). Bruxism physiology and pathology: An overview for clinicians. J. Oral. Rehabil..

[4] Sforza E., Jouny C., Ibanez V. (2000). Cardiac activation during arousal in humans: Further evidence for hierarchy in the arousal response. Clin. Neurophysiol..

[5] Huynh N., Kato T., Rompré P.H., Okura K., Saber M., Lanfranchi P.A., Montplaisir J.Y., Lavigne G.J. (2006). Sleep bruxism is associated to micro-arousals and an increase in cardiac sympathetic activity. J. Sleep. Res..

[6] Macaluso G.M., Guerra P., Di Giovanni G., Boselli M., Parrino L., Terzano M.G. (1998). Sleep bruxism is a disorder related to periodic arousals during sleep. J. Dent. Res..

[7] Kato T., Rompre P., Montplaisir J.Y., Sessle B.J., Lavigne G.J. (2001). Sleep bruxism: An oromotor activity secondary to micro-arousal. J. Dent. Res..

[8] Khoury S., Rouleau G.A., Rompre P.H., Mayer P., Montplaisir J., Lavigne G.J. (2008). A significant increase in breathing amplitude precedes sleep bruxism. Chest.

[9] Nashed A., Lanfranchi P., Rompré P., Carra M.C., Mayer P., Colombo R., Huynh N., Lavigne G. (2012). Sleep bruxism is associated with a rise in arterial blood pressure. Sleep.

[10] Yazıcıoğlu İ., Çiftçi V. (2021). Evaluation of signs and symptoms of temporomandibular disorders and incisal relationships among 7–10-year-old Turkish children with sleep bruxism: A cross-sectional study. Cranio.

[11] Smardz J., Martynowicz H., Michalek-Zrabkowska M., Wojakowska A., Mazur G., Winocur E., Wieckiewicz M. (2019). Sleep bruxism and occurrence of temporomandibular disorders-related pain: A polysomnographic study. Front. Neurol..

[12] Andrade de Alencar N., Nolasco Fernandes A.B., Gomes de Souza M.M., Luiz R.R., Fonseca-Gonçalves A., Maia L.C. (2017). Lifestyle and oral facial disorders associated with sleep bruxism in children. Cranio.

[13] Marpaung C., van Selms M.K., Lobbezoo F. (2018). Prevalence and risk indicators of pain-related temporomandibular disorders among Indonesian children and adolescents. Commun. Dent. Oral. Epidemiol..

[14] Rubin P.F., Erez A., Peretz B., Birenboim-Wilensky R., Winocur E. (2018). Prevalence of bruxism and temporomandibular disorders among orphans in southeast Uganda: A gender and age comparison. Cranio.

[15] Wieckiewicz M., Smardz J., Martynowicz H., Wojakowska A., Mazur G., Winocur E. (2020). Distribution of temporomandibular disorders among sleep bruxers and non-bruxers—A polysomnographic study. J. Oral. Rehabil..

[16] Lei J., Fu J., Yap A.U.J., Fu K.Y. (2016). Temporomandibular disorders symptoms in Asian adolescents and their association with sleep quality and psychological distress. Cranio.

[17] Topaloglu-Ak A., Kurtulmus H., Basa S., Sabuncuoglu O. (2022). Can sleeping habits be associated with sleep bruxism, temporomandibular disorders and dental caries among children?. Dent. Med. Probl..

[18] Cheifetz A.T., Osganian S.K., Allred E.M., Needleman H.L. (2005). Prevalence of bruxism and associated correlates in children as reported by parents. J. Dent. Child..

[19] Wieckiewicz M., Bogunia-Kubik K., Mazur G., Danel D., Smardz J., Wojakowska A., Poreba R., Dratwa M., Chaszczewska-Markowska M., Winocur E. (2020). Genetic basis of sleep bruxism and sleep apnea-response to a medical puzzle. Sci. Rep..

[20] Strasser B., Gostner J.M., Fuchs D. (2016). Mood, food, and cognition: Role of tryptophan and serotonin. Curr. Opin. Clin. Nutr. Metab. Care.

[21] Roberts K.M., Fitzpatrick P.F. (2013). Mechanisms of tryptophan and tyrosine hydroxylase. IUBMB Life.

[22] Jenkins T.A., Nguyen J.C., Polglaze K.E., Bertrand P.P. (2016). Influence of tryptophan and seroton in on mood and cognition with a possible role of the gut-brain axis. Nutrients.

[23] Minakuchi H., Fujisawa M., Abe Y., Iida T., Oki K., Okura K., Tanabe N., Nishiyama A. (2022). Managements of sleep bruxism in adult: A systematic review. Jpn. Dent. Sci. Rev..

[24] Cerón L., Pacheco M., Delgado Gaete A., Bravo Torres W., Astudillo Rubio D. (2023). Therapies for sleep bruxism in dentistry: A critical evaluation of systematic reviews. Dent. Med. Probl..

[25] Manfredini D., Ahlberg J., Winocur E., Lobbezoo F. (2015). Management of sleep bruxism in adults: A qualitative systematic literature review. J. Oral. Rehabil..

[26] Melo G., Duarte J., Pauletto P., Porporatti A.L., Stuginski-Barbosa J., Winocur E., Flores-Mir C., De Luca Canto G. (2019). Bruxism: An umbrella review of systematic reviews. J. Oral. Rehabil..

[27] Yap A.U., Chua A.P. (2016). Sleep bruxism: Current knowledge and contemporary management. J. Conserv. Dent..

[28] Mörmann W.H., Brandestini M., Mörmann W.H. (2006). State of the Art of CADS/CAM Restorations: 20 Years of CEREC.

[29] Pillai S., Upadhyay A., Khayambashi P., Farooq I., Sabri H., Tarar M., Lee K.T., Harb I., Zhou S., Wang Y. (2021). Dental 3D-Printing: Transferring Art from the Laboratories to the Clinics. Polymers.

[30] Duret F., Preston J.D. (1991). CAD/CAM imaging in dentistry. Curr. Opin. Dent..

[31] Blatz M.B., Conejo J. (2019). The Current State of Chairside Digital Dentistry and Materials. Dent. Clin. N. Am..

[32] Cunha T.M.A.D., Barbosa I.D.S., Palma K.K. (2021). Orthodontic digital workflow: Devices and clinical applications. Dental Press. J. Orthod..

[33] Joda T., Zarone F., Ferrari M. (2017). The complete digital workflow in fixed prosthodontics: A systematic review. BMC Oral. Health.

[34] Marcel R., Reinhard H., Andreas K. (2020). Accuracy of CAD/CAM-fabricated bite splints: Milling vs. 3D printing. Clin. Oral. Investig..

[35] Leib A.M. (2001). Patient preference for light-cured composite bite splint compared to heat-cured acrylic bite splint. J. Periodontol..

[36] Patzelt S.B.M., Krügel M., Wesemann C., Pieralli S., Nold J., Spies B.C., Vach K., Kohal R.J. (2022). In Vitro Time Efficiency, Fit, and Wear of Conventionally- versus Digitally-Fabricated Occlusal Splints. Materials.

[37] Zwarenstein M., Treweek S., Gagnier J.J., Altman D.G., Tunis S., Haynes B., Oxman A.D., Moher D. (2008). Improving the reporting of pragmatic trials: An extension of the CONSORT statement. BMJ.

[38] Casett E., Réus J.C., Stuginski-Barbosa J., Porporatti A.L., Carra M.C., Peres M.A., de Luca Canto G., Manfredini D. (2017). Validity of different tools to assess sleep bruxism: A meta-analysis. J. Oral. Rehabil..

[39] Castroflorio T., Mesin L., Tartaglia G.M., Sforza C., Farina D. (2013). Use of electromyographic and electrocardiographic signals to detect sleep bruxism episodes in a natural environment. IEEE J. Biomed. Health Inform..

[40] Castroflorio T., Deregibus A., Bargellini A., Debernardi C., Manfredini D. (2014). Detection of sleep bruxism: Comparison between an electromyographic and electrocardiographic portable holter and polysomnography. J. Oral. Rehabil..

[41] Deregibus A., Castroflorio T., Bargellini A., Debernardi C. (2014). Reliability of a portable device for the detection of sleep bruxism. Clin. Oral. Investig..

[42] Greene J.C., Vermillion J.R. (1964). The simplified oral hygiene index. J. Am. Dent. Assoc..

[43] Mayer P., Heinzer R., Lavigne G. (2016). Sleep Bruxism in Respiratory Medicine Practice. Chest.

[44] Vetter T.R. (2017). Fundamentals of Research Data and Variables: The Devil Is in the Details. Anesth. Analg..

[45] Breusch T.S., Pagan A.R. (1979). A Simple Test for Heteroscedasticity and Random Coefficient Variation. Econometrica.

[46] Durbin J., Watson G.S. (1971). Testing for Serial Correlation in Least Squares Regression. III. Biometrika.

[47] R Core Team R (2013). A Language and Environment for Statistical Computing.

[48] (2019). Regulation (EC) No 1394/2007 of the European Parliament and of the Council of 13 November 2007 on Advanced Therapy Medicinal Products and Amending Directive 2001/83/EC and Regulation (EC) No. 726/2004. https://ec.europa.eu/health//sites/health/files/files/eudralex/vol1/reg2007139/reg20071394en.pdf.

[49] Lo Giudice A., Ronsivalle V., Pedullà E., Rugeri M., Leonardi R. (2021). Digitally programmed (CAD) offset values for prototyped occlusal splints (CAM): Assessment of appliance-fitting using surface-based superimposition and deviation analysis. Int. J. Comput. Dent..

[50] Robinson P.G., Gibson B., Khan F.A., Birnbaum W. (2001). A comparison of OHIP 14 and OIDP as interviews and questionnaires. Commun. Dent. Health..

[51] (2015). Metallic materials. Instrumented indentation test for hardness and materials parameters. Part 1: Test Method.

[52] Oliver W.C., Pharr G.M. (1992). An improved technique for determining hardness and elastic modulus using load and displacement sensing indentation experiments. J. Mater. Res..

[53] Bargellini A., Graziano V., Cugliari G., Deregibus A., Castroflorio T. (2022). Effects on Sleep Bruxism Activity of Three Different Oral Appliances: One Year Longitudinal Cohort Study. Curr. Drug Deliv..

[54] Matsumoto H., Tsukiyama Y., Kuwatsuru R., Koyano K. (2015). The effect of intermittent use of occlusal splint devices on sleep bruxism: A 4-week observation with a portable electromyographic recording device. J. Oral. Rehabil..

[55] Lobbezoo F., Ahlberg J., Glaros A.G., Kato T., Koyano K., Lavigne G.J., de Leeuw R., Manfredini D., Svensson P., Winocur E. (2013). Bruxism defined and graded: An international consensus. J. Oral. Rehabil..

[56] Taira M., Maeda Y., Sawada T., Komatsu M., Kondo H. (2020). Mechanical properties of 3D-printed thermoplastic materials for orthodontic retainers. Dent. Mat. J..

[57] Monzavi A., Li W., Li Q., Swain M.V. (2018). Mechanical properties of 3D printed polymeric and ceramic orthodontic brackets. J. Mech. Behav. Biomed. Mat..

[58] Matinlinna J.P., Alomari S., Sadasivan M., Salehi H., Alagl A.S., Hamedani S. (2020). Effect of material properties and manufacturing process of occlusal splints on wear resistance: A literature review. J. Prosth. Dent..

[59] Cheah C.M., Chua C.K., Tan K.H., Abu Bakar M.S. (2019). Evaluation of mechanical properties and dimensional accuracy of 3D-printed occlusal splints. Int. J. Prosth..

[60] Hwang Y.H., Song J.H., Hong STKim H.Y. (2021). Evaluation of the mechanical properties of 3D-printed occlusal splints made of biocompatible resin. Materials.

[61] Li W., Bai S., Zhou J., Xu X., Wang Y. (2020). Comparison of mechanical properties between 3D-printed and traditionally polymerized occlusal splint materials. J. Prosth. Dent..

[62] Nayyer M., Savabi O. (2020). Wear comparison of CAD/CAM milled and 3D printed occlusal splints. J. Prosth. Dent..

[63] Paradowska-Stolarz A., Wezgowiec J., Malysa A., Wieckiewicz M. (2023). Effects of Polishing and Artificial Aging on Mechanical Properties of Dental LT Clear^®^ Resin. J. Funct. Biomater..

[64] Raphael K.G., Janal M.N., Sirois D.A., Dubrovsky B., Wigren P.E., Klausner J.J., Krieger A.C., Lavigne G.J. (2013). Masticatory muscle sleep background electromyographic activity is elevated in myofascial temporomandibular disorder patients. J. Oral. Rehabil..

[65] Greene C., Manfredini D., Ohrbach R. (2023). Creating patients: How technology and measurement approaches are misused in diagnosis and convert healthy individuals into TMD patients. Front. Dent. Med..

